# A Comparative Study of Rank Aggregation Methods in Recommendation Systems

**DOI:** 10.3390/e25010132

**Published:** 2023-01-09

**Authors:** Michał Bałchanowski, Urszula Boryczka

**Affiliations:** Institute of Computer Science, Faculty of Science and Technology, University of Silesia in Katowice, Będzińska 39, 41-200 Sosnowiec, Poland

**Keywords:** recommendation systems, rank aggregation, rank fusion

## Abstract

The aim of a recommender system is to suggest to the user certain products or services that most likely will interest them. Within the context of personalized recommender systems, a number of algorithms have been suggested to generate a ranking of items tailored to individual user preferences. However, these algorithms do not generate identical recommendations, and for this reason it has been suggested in the literature that the results of these algorithms can be combined using aggregation techniques, hoping that this will translate into an improvement in the quality of the final recommendation. In order to see which of these techniques increase the quality of recommendations to the greatest extent, the authors of this publication conducted experiments in which they considered five recommendation algorithms and 20 aggregation methods. The research was carried out on the popular and publicly available MovieLens 100k and MovieLens 1M datasets, and the results were confirmed by statistical tests.

## 1. Introduction

With the number of products and services available today, making favorable consumer decisions is ever more difficult. Users, trying to cope with this, often read reviews and comments available online, hoping that they will help them make the right choice. Unfortunately, acquiring relevant information from such a large amount of data is often very difficult and time-consuming [1]. In addition, there are also issues related to the validity and reliability of the very data from which conclusions are drawn [2].

In order to solve this problem, recommender systems have been proposed that assist the user in the decision-making process by suggesting products or services that are most likely to be of interest to him [3]. Over the years, they have grown greatly in popularity and are now often an integral part of social media platforms and auction sites. While the idea behind these systems is relatively simple, they can be very complicated and hard to implement, as they often integrate data that comes from various sources [4].

In the literature, the problem of recommendations is presented as a problem of predicting the rating that a user would give to a given item [5], or as a problem of predicting the ranking of items that would be suggested to the user [6]. Undoubtedly, the second approach is closer to the actual application of recommender systems, where usually the results of such systems are presented to the user in the form of a ranking [7]. However, it should be noted that users are more likely to select content in the first positions of such a ranking than in the last positions. For this reason, dedicated measures are used to evaluate each of these approaches, which are used to determine the accuracy of the generated recommendations [8]. It is also worth mentioning that recommendation accuracy is not the only criterion that we can use to evaluate the effectiveness of such systems. Other measures that have also been proposed in the literature include novelty and diversity [9].

Over the past few years, a number of algorithms have been proposed that are designed to generate recommendations in the form of a ranking (called “TopN recommendation algorithms”) [10]. Despite years of research, no universal algorithm has been proposed to generate high-quality recommendations in all cases. In addition, when we compare the generated recommendations in the context of a particular user, these algorithms do not generate identical recommendations. For this reason, the literature suggests the use of aggregation methods, the task of whose is to aggregate the rankings, generated by individual recommendation algorithms, in order to create a new, “better” recommendation.

For example, in the book [11] [p. 417], the author indicated that this is a problem that has not yet been sufficiently studied in the context of recommender systems and is an interesting direction for future research. Similar conclusions were presented in the publication [12], where the authors pointed out that relatively few dedicated algorithms have been developed in the literature related to recommender systems that address this problem. Therefore, the authors of this publication recognize a research gap in this area, related to the study of rank aggregation methods in the context of recommendation systems.

The main contribution of this paper is to investigate which classical aggregation methods, based on supervised and unsupervised learning, produce the best aggregation. To allow easy reproduction of the experimental results, popular datasets were used for the experiments. Although in the literature there are publications that have already analyzed this problem in the context of recommender systems [12], in this paper, experiments are conducted on a bigger number of classical aggregation methods. It is important since these techniques are often used as comparative methods and presented together with the results of new algorithms proposed by researchers. This paper aims to help researchers decide which aggregation methods are worth considering when reporting the results of their experiments in the context of recommender systems.

In addition, the authors are also aware of the problem of reproducibility of experimental results that are reported in scientific publications. In the context of recommender systems, this problem has already been pointed out many times [13,14,15,16], emphasizing that due to the complexity of recommender systems and the different methods of their evaluation, reproducing the results of experiments without access to the source code is often very difficult and sometimes even impossible. With this in mind, the authors of this publication provide the research environment, which was created for the purpose of conducting experiments. It was implemented in the Python programming language, based on publicly available programming libraries.

The article has been divided into six chapters. Section 2 presents a literature review, referring mainly to the problem of rank aggregation in recommender systems. Section 3 presents a formal definition of recommendation system and rank aggregation problem. Section 4 presents details related to the research environment used, and details of the parameter tuning process. In addition, in this chapter, metrics for evaluating the quality of the generated recommendations and the evaluation protocol will be discussed. In Section 5, the results of the experiments are presented with appropriate commentary. Section 6 is dedicated to conclusions and suggestions for future work.

## 2. Related Works

The problem of rank aggregation is a well known, especially in the context of social choice theory, which deals with the analysis of collective decision-making and how to transform the preferences of individual users into the preferences of the group [17]. In the context of modern information filtering systems, the problem of rank aggregation was described by C. Dwork in his work [18], where the author presented the theoretical basis of this problem, analyzing it through the prism of information retrieval systems. In the following years, applications of this idea have been proposed in other areas of science, which are related to: combining microarray data [19], similarity search and object classification [20], and biology [21].

Within the context of recommender systems, there has been relatively little work related to this problem, and as noted in [11] (p. 417), it is a relatively under-researched field. However, it is hard to say clearly when the idea was first used in recommender systems, since it is not always defined clearly as a “rank aggregation problem” in scientific publications. It seems that the first papers using this concept were works related to hybrid systems [22,23].

Rank aggregation is primarily used in the generation of group recommendations. Group recommendations, unlike their classical counterparts, generate recommendations that are tailored to the preferences of an entire group of users, and not just to one specific user [24]. One such system is [25], where the authors presented interesting results, suggesting that for some users, group recommendations may prove better, than personalized recommendations. In the publication [26], the authors proposed an aggregation algorithm based on the Borda method. In turn, in the paper [27], the authors suggested using entropy to analyze the distribution of ratings and detect items on which group members did not reach consensus.

The rank aggregation problem is a computationally expensive problem and it has been proven that from a certain number of rankings it becomes an NP-hard problem [18,28]. Therefore, an interesting direction of research is the use of metaheuristic algorithms that allow finding an approximate solution in an acceptable time. For example, in the publication [29], the authors proposed a hybridization technique that combines recommendations generated by different recommendation algorithms, using an evolutionary algorithm for multi-criteria optimization. The publication [30] suggested an Evolutionary Rank Aggregation (ERA) algorithm that used genetic programming to directly optimize the MAP measure. The authors tested the suggested solution on four datasets, and the results clearly indicate that the technique improved the quality of the generated recommendations. In another publication [31], the authors proposed the Multi-objective Evolutionary Rank Aggregation (MERA) algorithm, which was an algorithm for multi-criteria optimization. The publication [32] suggested using the Differential Evolution algorithm, to directly optimize the AP measure for individual users in the system. This approach made it possible to find a vector, determining the preference of a given user over individual rankings. However, the main disadvantage of techniques based on metaheuristic algorithms is that, they are often difficult to implement correctly and require appropriate tuning.

What is particularly noteworthy is the publication of [12], in which the authors tried to answer the question of whether the use of rank aggregation methods in recommender systems can be effective. To this end, they conducted a systematic study in which they considered as many as 15 recommendation algorithms and 19 aggregation methods, and the experiments were carried out on seven different datasets. Analyzing the results of the study, the authors found that aggregation techniques improved the quality of recommendations on the six tested datasets.

## 3. Background of the Research

This chapter presents the basic concepts, related to the subject of this article. First, the basic information on recommender systems is discussed, and then the problem of rank aggregation within the context of these systems is presented.

### 3.1. Recommender Systems

The task of a recommender system is, on the basis of historical data, to predict the future preferences of users. Nowadays, they are increasingly used in various areas of our lives, from buying items on auction websites, through choosing the next movie to watch, to adding new friends on social media. However, this is not a trivial problem, and intensive research work has been carried out on the subject for many years now [33]. The most important event that significantly increased interest in this problem was a competition organized by Netflix, where researchers who managed to sufficiently increase the quality of recommendations generated were offered 1 million dollars as a prize [34].

In recommender systems, we can distinguish two main approaches to generating recommendations. They can be based on an attempt to predict what rating (e.g., on a scale of 1 to 5) a user would give to a given item in the system [35]. They may also try to predict a certain set of items, most often presented as an ordered list, that would be recommended to the user [6].

Recommender systems can be also divided into personalized and non-personalized. A non-personalized recommender system is one that, based on the global behavior of all users in the system, tries to draw some conclusions, for example, recommending to the user the movies that are most watched. Nowadays, however, personalized systems are mostly used, which, based on the historical activity of a given user, create a profile of that user, which is used to generate recommendations [36].

Formally, in a recommender system, we distinguish a certain set of users $U=\{u_{1},\;\ldots ,\;u_{\left|U\right|}\}$ and a certain set of items $I=\{i_{1},\;\ldots ,\;i_{\left|I\right|}\}$. All interactions between users and items are recorded in a matrix *R*. Thus, the data can be represented as a triple $\left(u,\;i,\;r_{ui}\right)$, which means that a given user u∈U interacted with an item i∈I, giving it a rating $r_{ui}$. All ratings given by users to items are often represented as a user–item interaction matrix *R*.

In the context of recommender systems, many techniques and methods have been proposed, and for this reason, the literature has suggested dividing them into the following approaches: content-based filtering [37], collaborative filtering [38], knowledge-based filtering [39] and a combination of different techniques, a hybrid approach [40]. One of the most popular techniques for generating recommendations is the matrix factorization technique [41], which transforms the matrix *R* into two smaller matrices according to the following formula:

(1) $$R\approx PQ^{T}.$$

**Figure 1:**
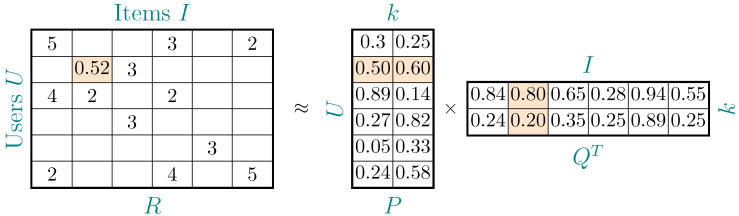
An example of user–item matrix factorization.

In this decomposition, *P* is a matrix representing user features of |U|×k, and *Q* is a matrix representing item features of |I|×k. Then, in order to determine the user’s preference *u* for an item *i*, it is required to:

(2) $$s\left(i|u\right)={\vec{p}}_{u}\;\cdot \;{\vec{q}}_{i},$$

where ${\vec{p}}_{u}$ is the feature vector for user *u*, and ${\vec{q}}_{i}$ is the feature vector for item *i*. This technique allows users and items to be represented by a small number of latent features, and it has become very popular due to Simon Funk [42], who used it in the Netflix competition. An example of such factorization is presented below in Figure 1, along with Table 1 showing the recommendation algorithms used in the experimental phase.

### 3.2. Rank Aggregation

The rank aggregation problem refers to the situation where, having several rankings, which are ordered lists consisting of certain objects (e.g., items), our task is to create a new ranking that is “better” than the base rankings.

Formally, this problem can be presented as follows. Let us assume that we have a certain set of elements $I=\{i_{1},i_{2},\cdots ,i_{m}\}$. We define a ranking as an ordered list of these elements $\tau =[i_{j}\geq i_{h}\geq \cdots \geq i_{z}]$, where ≥ denotes the order relation between the elements in the set *I*, and the relevance of an element is determined by its position. With the symbol $\tau (i_{j})$, the position (or rank) of item $i_{j}$ in the τ ranking will be denoted. Two items $i_{j}$ and $i_{h}$ can be compared using their positions in ranking τ. For example, we can say that item $i_{j}$ is in a “better” position than item $i_{h}$, which will be denoted as $\tau \left(i_{j}\right)<\tau \left(i_{h}\right)$. In addition, a single algorithm will be denoted as $a_{h}$, and the set of all algorithms will be denoted as $A=\{a_{1},a_{2},\cdots ,\;a_{n}\}$. Each algorithm generates a ranking τ, and the set of all rankings will be denoted as $T=\{\tau_{1},\tau_{2},\cdots ,\tau_{n}\}$, where *n* denotes the number of algorithms and the number of generated rankings.

The goal of rank aggregation is to create a new ranking $\tau^{*}$, which in theory should be better than the individual rankings in the set *T*. The quality of a ranking should be considered in the context of a given problem, keeping in mind its specifics. For example, in recommender systems, this could mean the ranking that most improves the quality of the recommendation, where this quality can be calculated based on the measures described in Section 4.3. For unsupervised methods, however, dedicated distance measures are more often used to determine the degree of similarity between rankings (e.g., Kendall Tau distance [46]).

Therefore, the problem of rank aggregation boils down to defining an aggregate function Ψ that, based on the rankings in the set *T*, generates a new ranking $\tau^{*}$:

(3) $$\Psi :\left\{\tau_{1},\tau_{2},...,\tau_{n}\right\}\to \tau^{*}.$$

Depending on the available data, the Ψ aggregate function can be created based on different methods. In the literature, the basic division of these methods is by score-based and permutation-based methods. With score-based methods, each element in the ranking is assigned a certain value, which determines its position in the ranking. Aggregation methods then create a new ranking $\tau^{*}$ by combining the scores from the base rankings. Permutation-based rank aggregation methods, on the other hand, create an aggregation by searching the entire space for possible permutations of elements from the set *I*.

Aggregation methods can also be divided, based on the type of learning algorithm used. Methods based on supervised learning [47] create a rank model using a training set. More advanced techniques may also use an approach called “learning to rank” [48], but they are much more complex and difficult to implement, although they can obtain better results compared to other methods [12]. In techniques based on unsupervised learning, aggregation is most often created based on dedicated distance measures that allow individual rankings to be compared with each other (e.g., Kendall Tau distance) [46]. These methods are characterized by the simplicity of implementation and the fact that they do not need a training phase to operate.

**Figure 2:**
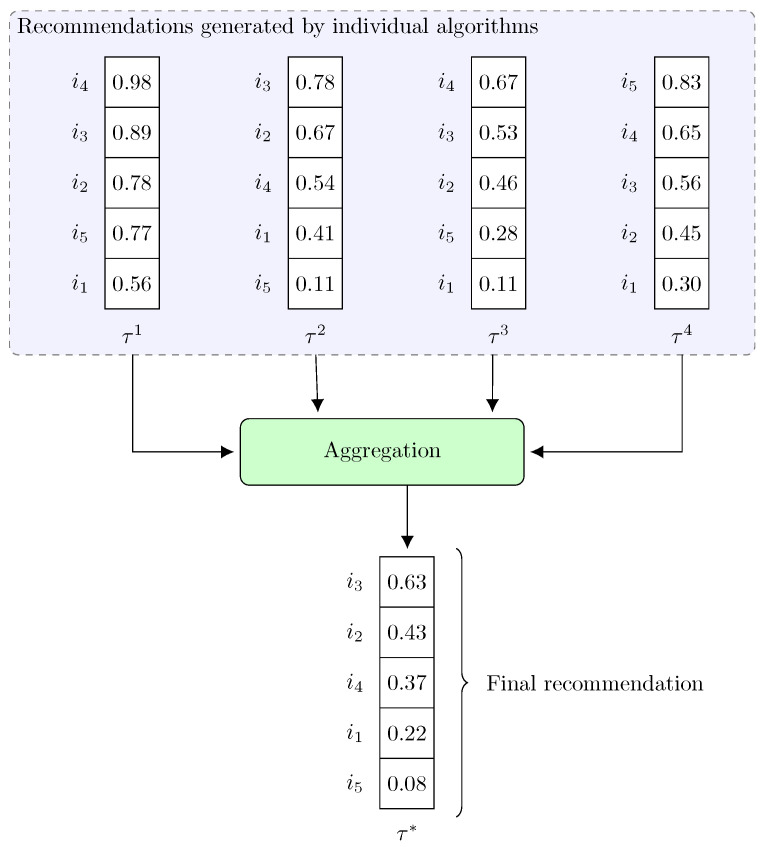
An overview figure showing an exemplary aggregation of 4 rankings. Each recommendation algorithm generates a recommendation, assigning a certain score to each item in the system. An aggregation method is then used, which combines the rankings to form the final recommendation $\tau^{*}$.

Over the years, a number of different techniques have been suggested in the literature that can be used to create rank aggregation, and an overview of them is presented in the publications [12,49]. Below is an overview Figure 2 showing the aggregation process of four rankings that were generated by four recommendation algorithms. In turn, in the Table 2, a summary of the aggregation methods used in the experimental phase is presented.

## 4. Experimental Evaluation

This chapter discusses the details of the process of conducting the research. First, the experimental setting used to conduct the experiments will be presented. Then, the process of tuning the parameters of the recommendation algorithms is discussed, and the measures used to evaluate the quality of the generated recommendations are presented. Finally, the evaluation protocol is discussed.

### 4.1. Experimental Setup

To make it possible to conduct the research, it was necessary to prepare a dedicated RecRankAgg research environment, since none of the existing solutions met the necessary requirements. In addition, to reduce the time needed for implementation and the chances of possible errors, existing programming libraries that already had some of the needed functionality were used.

The recommender system was created based on the LensKit [43] library, which is a library that is a set of tools designed to conduct research work related to recommender systems. It has numerous functionalities, which include: loading and dividing the dataset into training and test sets, evaluating the generated recommendations using various quality measures. In addition, some implementations of recommendation algorithms are available in this library, which are presented in Table 1. The aggregation methods presented in Table 2 and used in the experimental phase, were available in the Ranx library [60,61]. The RecRankAgg experimental setting was implemented in Python, and the experiments were conducted on an Intel Core i5-7600 (3.50 GHz) computer with 16 GB RAM.

The research was conducted on two popular datasets MovieLens 100k and MovieLens 1M [62]. The number in the name of this dataset indicates the number of available ratings. The choice of smaller versions of the datasets was motivated by the fact that as the number of available ratings increased, so did the time required to train the various models of recommendation algorithms, which also lengthened the process of tuning hyperparameters. These are popular and publicly available datasets from which the results of the experiments can be easily reproduced. The MovieLens 100k dataset contains 100,000 ratings by 943 users for 1682 movies. Each user in this dataset rated at least 20 movies, on a scale of 1 to 5. By contrast, the MovieLens 1M dataset contains 100,000,209 ratings, which were given by 6040 users for 3952 movies. Similarly, as with the smaller version of this dataset, it has been cleaned up properly beforehand, and users who have rated fewer than 20 movies have been removed from it. In addition, all ratings issued in these datasets have a timestamp.

### 4.2. Parameters Tuning

Before generating recommendations, the parameters of the recommendation algorithms must be properly tuned. The goal of this process is to find a set of parameters that maximizes the quality of the generated recommendations (expressed using the MAP measure), using a training set (60%) and a validation set (20%).

The software used to tune the parameters, was the Optuna library [63], which allows automation of this process. The tuning algorithm was Tree-structured Parzen Estimator [64], which creates a probabilistic model based on the history of previous hyperparameter values, and then uses it to suggest subsequent hyperparameter values. To keep the tuning process from being too long, a trial limit of 100 was set in advance.

Table 3 presents the parameters of the recommendation algorithms, along with their type, range of values, and the best value that was found during the tuning process. The names of the tuned parameters used are consistent with the parameter names available in the LensKit library. The results of the tuning process are presented in the form of graphs in the Appendix A and will be discussed below.

**Figure A1:**
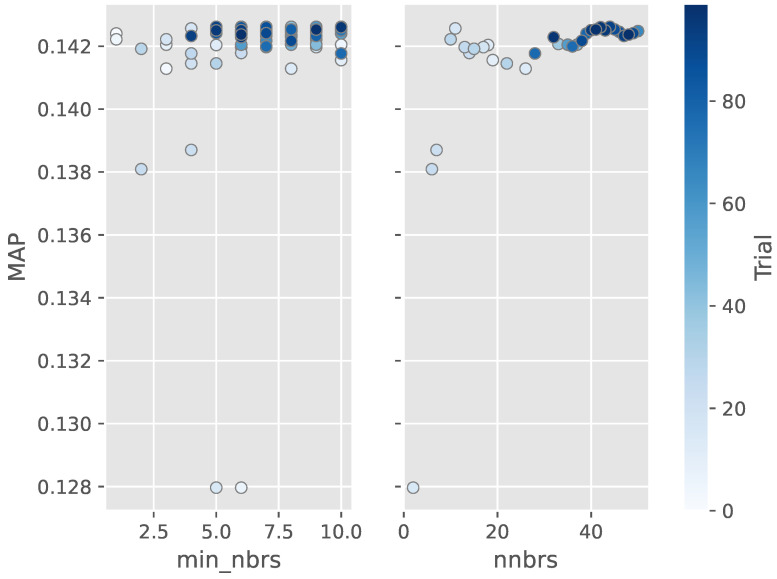
Tuning of parameters: min_nbrs and nnbrs for ItemkNN algorithm on MovieLens 100k dataset. The figure represents how a change in the value of these parameters affects the quality of the generated recommendations, expressed by the MAP measure.

Figure A1 shows the process of tuning the min_nbrs and nnbrs parameters of the Item kNN algorithm. It can be noticed that the min_nbrs parameter was unlikely to affect the quality of the generated recommendations, since it obtained high MAP values, practically for the entire range of values taken. By contrast, the nnbrs parameter affected the quality of recommendations to the greatest extent, especially when it took values in the range [15,35].

**Figure A2:**
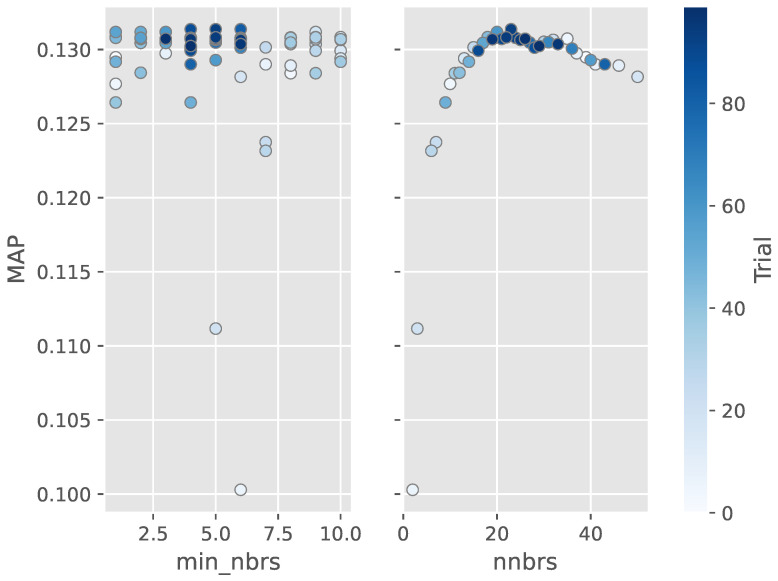
Tuning of parameters: min_nbrs and nnbrs for UserkNN algorithm on MovieLens 100k dataset. The figure represents how a change in the value of these parameters affects the quality of the generated recommendations, expressed by the MAP measure.

Similar results of the parameter tuning process were obtained for the User kNN algorithm, as can be seen by analyzing the Figure A2. In the case of this algorithm, the min_nbrs parameter also did not significantly affect the quality of the results, while for the nnbrs parameter, the quality of recommendations was greatest when this parameter took values in the range [35,50].

**Figure A3:**
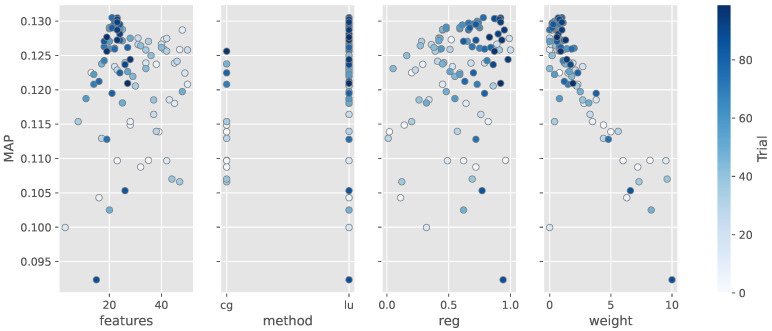
Tuning of parameters: features, method, reg and weight for ImplicitMF algorithm on MovieLens 100k dataset. The figure represents how a change in the value of these parameters affects the quality of the generated recommendations, expressed by the MAP measure.

For the ImplicitMF algorithm, four parameters were tuned: features, method, reg and weight. The results of this process are shown in Figure A3. For the features parameter, the quality of the recommendation was highest when this parameter took values in the range [20,25]. The method for which the algorithm achieved the best results was the cg method. For the reg parameter, the quality of recommendations was greatest when the value of the parameter was close to the value of 0.8. In addition, large values of the weight parameter significantly affected the quality of the generated recommendations, because as the value of this parameter increased, the quality of the generated recommendations also decreased, and the optimal value turned out to be 1.

**Figure A4:**
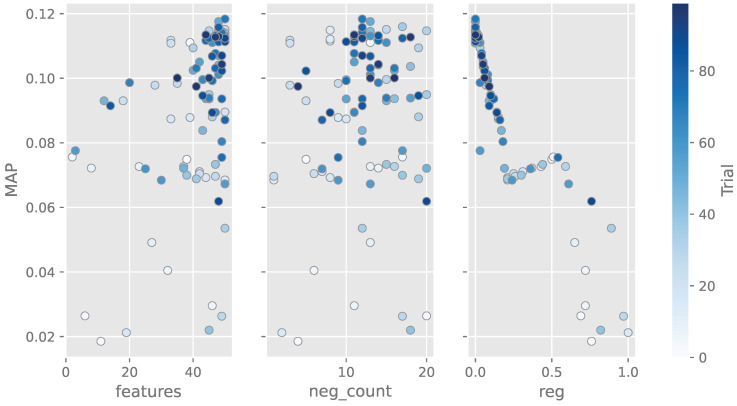
Tuning of parameters: features, neg_count and reg for BPR algorithm on MovieLens 100k dataset. The figure represents how a change in the value of these parameters affects the quality of the generated recommendations, expressed by the MAP measure.

The last of the algorithms to be tuned was the BPR algorithm. Analyzing the Figure A4, it can be seen that when the features parameter took a value close to the value of 45, the quality of the generated recommendations was the highest. In addition, the neg_count parameter should take values above 10, and as the value of the reg parameter increased, the quality of the generated recommendations significantly decreased, and the best value, turned out to be 0.

### 4.3. Evaluation Metrics

To evaluate aggregation methods, dedicated measures will be used to determine the quality of the created ranking. These measures compare the generated recommendations with the items that are in a given user’s test set. The most basic measures that can be used for this purpose are precision and recall. Precision represents the percentage of relevant items that appeared in the recommended ranking, while recall represents the percentage of relevant items that were recommended. These measures are calculated according to the following formulas:

(4) $$Precision@k\left(\tau_{i}^{r}\right)=\frac{|Rel\left(u_{i}\right)\cap \tau_{i}^{r}@k|}{|\tau_{i}^{r}@k|},$$

(5) $$Recall@k\left(\tau_{i}^{r}\right)=\frac{|Rel\left(u_{i}\right)\cap \tau_{i}^{r}@k|}{\left|Rel(u_{i})\right|},$$

where $Rel(u_{i})$ is the set of relevant items for user $u_{i}$, and $\tau_{i}^{r}@k$ denotes the first *k* items in the ranking where the recommended items are located.

The fundamental disadvantage of simple precision is that it does not take into account the position in which the relevant items are located. For this reason, to assess the quality of recommendations, an AP measure is used, which averages the precision values calculated for each item in the recommended ranking, according to the following formula:

(6) $$AP@k\left(\tau_{i}^{r}\right)=\sum_{z=1}^{K}\frac{Precision@z\left(\tau_{i}^{r}\right)\times rel_{u_{i}}\left(x_{z}\right)}{min(|Rel\left(u_{i}\right)|,|r_{i}^{r}|)},$$

where $rel_{u_{i}}\left(x_{z}\right)$ determines the relevancy of an item $x_{z}$ to a user $u_{i}$. The advantage of this measure is that it penalizes incorrect ordering of items in the ranking.

The average precision described above is usually used when evaluating recommendations in the context of a single user. However, we often want a single number as the result of our experiments. Therefore, a mean average precision was suggested, expressed by the means of the following formula:

(7) $$MAP@k=\frac{1}{\left|U\right|}\sum_{i=1}^{\left|U\right|}AP@k\left(\tau_{i}\right).$$

AP and MAP measures are often used for binary values, but in a situation where there are different levels of relevance in the system and we have information on how relevant an item is (e.g., on a scale of 1 to 5), it makes sense to use a measure of normalized discounted cumulative gain. As with the MAP measure, the purpose of this measure is to reward items that are high (closer to the first position) on the recommended list, as expressed by the following formula:

(8) $$DCG\left(\tau_{i}^{r}\right)=\sum_{x_{j}\in \tau_{i}^{r}}\frac{rel_{u_{i}}\left(x_{j}\right)}{\log_{2}\left(\tau_{i}^{r}\left(x_{j}\right)+1\right)}.$$

The measure of DCG cannot be compared between users, since each user has a different number of relevant items. For this reason, a normalization is performed that uses the ideal discounted cumulative gain IDCG, which determines the maximum value of DCG for a ranking of $\tau_{i}^{r}$. Then, to obtain the NDCG measure, it is necessary to:

(9) $$NDCG\left(\tau_{i}^{r}\right)=\frac{DCG(\tau_{i}^{r})}{IDCG(\tau_{i}^{r})}.$$

### 4.4. Evaluation Protocol

For the experiments, the recommendation algorithms presented in the Table 1 were used. Each of the algorithms $A=\{a_{1},a_{2},\cdots ,a_{n}\}$, generates a ranking of τ and each user $u_{i}$ is represented by a collection of rankings $T=\{\tau_{1},\tau_{2},\cdots ,\tau_{n}\}$. Recommendation algorithms generate recommendations using the parameters found during the parameter tuning process described in Section 4.2.

To carry out the evaluation process, the dataset had to be properly prepared. First, user ratings were sorted by timestamp. This approach is justified [16] (p. 46) because our task is to predict the future choices of users, based on their previous activity.

Then, for each user, the items he rated were divided into three sets: training (60%), validation (20%) and test (20%). The training and validation sets were used for the process of tuning the parameters of the recommendation algorithms and aggregation methods. However, it should be noted that during the final evaluation, the training set is combined with the validation set, so the division of the sets is as follows: training set (80%) and test set (20%).

Each recommendation algorithm generated recommendations in the form of ranking of 10 items. The following measures were used for evaluation: NDCG@10, MAP@10, P@1, P@10, and Recall@10. In addition, in order to demonstrate the statistical significance of the presented results, a Fisher’s randomization test was performed, and the symbol 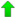 was used to indicate that a particular aggregation method, obtained statistically significant results (with 95% certainty), compared to all recommendation algorithms used in the experiments. The choice of this statistical test is consistent with the suggestions for the evaluation process of information filtering systems found in the literature [65].

## 5. Results

This chapter presents the results of experiments that were carried out using RecRankAgg software. Due to the fact that this research was carried out on two datasets, this chapter is divided into two subsections corresponding to each dataset.

### 5.1. Results on MovieLens 100k

Analyzing the results presented in Table 4, it can be seen that the non-personalized recommendation algorithm, Most Popular, generated recommendations that were clearly inferior, taking into account all the measures that were used to assess the quality of recommendations. Such a result, however, is not surprising, as mostly non-personalized recommendation algorithms perform worse than personalized algorithms. The personalized algorithm that achieved the best results was the ImplicitMF algorithm. However, it should be noted that the differences in the quality of the generated recommendations, between the personalized recommendation algorithms used in the experiments, are relatively small.

Analyzing the effectiveness of unsupervised aggregation methods, it can be noted that statistically significant results, due to the NDCG@10 measure, were achieved by the following methods: CombMNZ, Bordafuse, LogISR. On the other hand, the methods of this type that formed the lowest quality aggregation were: CombMIN, CombMED, and CombANZ.

Supervised aggregation methods mostly achieved statistically significant results for the NDCG@10 measure, while for the P@10 and MAP@10 measures, statistically significant results were achieved by: Slidefuse, Bayesfuse and Posfuse. The methods that achieved the worst results were: Weighted Sum and Weighted Borda. It should also be noted that the supervised LognISR algorithm, achieved an identical result as the unsupervised LogISR algorithm.

Analyzing the results of the study, it is worth noting the results obtained by the various methods for the P@1 measure. This measure determines the precision, taking into account only item in the first position in ranking. Some of the aggregation methods (e.g., LogISR, ISR, LognISR), perform noticeably better at correctly positioning items that are in this position.

### 5.2. Results on MovieLens 1M

Analyzing the results of the experiments presented in Table 5, which were carried out on the MovieLens 1M dataset, it can be seen that, as with the MovieLens 100k dataset, the non-personalized Most Popular recommendation algorithm generated recommendations of significantly lower quality than other recommendation algorithms. The personalized algorithm that achieved the best results was the Item kNN algorithm. The differences in the quality of the generated recommendations between the neighborhood-based algorithms (User kNN and Item kNN) were insignificant. The same situation occurred for algorithms based on matrix factorization (ImplicitMF and BPR).

Analyzing the effectiveness of the unsupervised aggregation methods, it can be noted that due to the NDCG@10 measure, the methods that reached statistical significance are: LogISR, Bordafuse, CombSUM, CombMNZ and ISR. For this dataset, the aggregation methods that generated the lowest quality aggregation were: CombMIN, CombMED, CombANZ and Condorcet. Therefore, it can be seen that for both datasets, virtually the same methods created low-quality aggregations.

Almost all of the supervised methods achieved statistically significant results for the NDCG@10 measure and MAP@10. For this dataset, it is also noteworthy that some of the unsupervised methods achieved results similar, to the supervised methods.

## 6. Conclusions

This article presents the results of our research, the aim of which was to test the effectiveness of aggregation methods in recommendation systems. Five recommendation algorithms and 20 aggregation methods (10 supervised methods and 10 unsupervised methods) were used to conduct it. The process of parameter tuning was also discussed and the RecRankAgg experimental environment was provided for easy reproduction of the performed experiments. In addition publicly available MovieLens 100k and MovieLens 1M datasets were used in the study.

The results of the experiments were confirmed by statistical tests and clearly indicate that aggregation methods can be successfully used in the context of recommender systems. However, it should be noted that their effectiveness varies. In general, better results can be obtained using supervised algorithms, but interestingly, some unsupervised techniques obtained results similar to supervised techniques. It is a very interesting observation and we intend to conduct a more detailed analysis of such cases in the future. In addition, it is important to keep in mind that the parameters of the recommendation algorithms should be properly tuned before creating aggregations, since, as presented in Section 4.2, the influence of individual parameters varies greatly.

To help researchers choose which aggregation algorithms to consider when reporting experimental results, the following recommendations have been made:

- Based on the analysis in Section 5.1, when reporting experimental results on the MovieLens 100k dataset, it is worth considering the following unsupervised aggregation methods: LogISR, Bordafuse and CombMNZ. On the other hand, among the supervised aggregation methods, it is worth considering: RRF, Slidefuse, Bayesfuse, RBC, LognISR, and Posfuse.
- Based on the analysis in Section 5.2, when reporting the results of experiments on the MovieLens 1M dataset, it is worth considering the following unsupervised aggregation methods: LogISR, Bordafuse, and CombMNZ. On the other hand, for the supervised aggregation methods, practically all the methods used gave statistically significant results for NDCG@10 and P@10 measures.

Although a direct comparison of the results obtained in this article with the results presented in the publication [12] is not possible (because a different number of recommendation algorithms were used), it is possible to notice some interesting similarities in the presented results. For example, when analyzing the results of experiments on the MovieLens 1M dataset (presented in the online appendix of this publication [12]), it can be seen that some supervised algorithms obtained results similar to unsupervised algorithms. This observation coincides with the results presented in our paper. The authors in [12] noted that supervised algorithms could generate aggregations of higher quality than unsupervised ones when they have access to diverse rankings. However, a closer analysis of such cases seems reasonable.

When comparing the results of experiments, it should also be noted that the problem of rank aggregation is quite complex since the aggregation is performed based on previously generated rankings. Although a dedicated research environment RecRankAgg has been prepared for this paper, which significantly facilitates the reproduction of the experiments performed, it is also a good idea to create ready-to-use datasets with previously generated rankings in the future. It would provide an easy way to conduct and reproduce the results of the experiments for other researchers.

In the future, we intend to use more diverse datasets and include more recommendation algorithms in our research. It is also an interesting direction for future work to see which aggregation algorithms perform best with low-quality rankings. It can be done by including in the aggregation process algorithms that generate recommendations of very low quality (e.g., random recommendations). Another interesting direction of research is to see how different variants of the normalization process, affect the quality of the created aggregation. In addition, in the research conducted, the recommendation algorithms generated rankings that consisted of only 10 items. It would also be reasonable to check how the number of items that are recommended by different recommendation algorithms affects the quality of the created aggregation.

Another interesting direction for future research is to consider the problem of rank aggregation from the perspective of consensus theory. For example, in the literature, some papers propose a dedicated measure for calculating the consensus between rankings [66]. Attention is also paid to the problem of the so-called “fair consensus” [67], in which it is considered whether aggregation can introduce disadvantageous bias to particular groups. Dedicated algorithms have been proposed to solve this problem, and their effectiveness has been tested on real-world datasets [68].

## Figures and Tables

**Table 1:** Selected recommendation algorithms available in the LensKit [43] library that were used in the experimental phase.

Algorithm	Type	Description	Reference
UserKNN	Neighborhood based	User-user nearest-neighbor collaborative filtering	[43]
ItemKNN	Neighborhood based	Item-item nearest-neighbor collaborative filtering	[6]
BPR	Matrix factorization	Bayesian Personalized Ranking with matrix factorization, optimized with TensorFlow	[44]
ImplicitMF	Matrix factorization	Implicit matrix factorization trained with alternating least squares (ALS)	[45]
MostPopular	Non-personalized	Recommend the most popular items	[43]

**Table 2:** Selected aggregation methods used in the experimental phase.

Aggregation Method	Method Type	Reference
CombMIN	Unsupervised	[50]
CombMED		[50]
CombANZ		[50]
LogISR		[51]
Bordafuse		[52]
Condorcet		[53]
CombMAX		[50]
CombSUM		[50]
CombMNZ		[50]
ISR		[51]
CombGMNZ	Supervised	[54]
RRF		[55]
Slidefuse		[56]
Bayesfuse		[52]
WMNZ		[57]
RBC		[58]
LognISR		[51]
Posfuse		[59]
Weighted Sum		[50]
Weighted Borda		[52]

**Table 3:** The parameters of the recommendation algorithms, along with the type and range of values that were used during the parameter tuning process. The table also presents the best value found.

Algorithm	Parameter	Range	Data Type	Best Value
UserKNN	nnbrs	[2–50]	int	23
	min_nbrs	[0–10]	int	4
ItemKNN	nnbrs	[2–50]	int	44
	min_nbrs	[0–10]	int	7
BPR	num factors	[2–50]	int	50
	reg	[0–1]	double	0
	neg count	[0–20]	int	12
ImplicitMF	num factors	[2–50]	int	21
	method	cg, lu	categorical	lu
	reg	[0–1]	double	0.78
	weight	[0–10]	double	1
MostPopular	topN		int	10

**Table 4:** The results of the experiments conducted on the MovieLens 100k dataset, representing the quality of recommendations generated by individual recommendation algorithms and aggregation methods.

Type	Algorithm Name	NDCG@10	MAP@10	P@1	P@10	Recall@10
Recommendation algorithms	BPR	0.174	0.053	0.222	0.163	0.116
	ImplicitMF	0.181	0.059	0.230	0.166	0.119
	Item kNN	0.181	0.058	0.230	0.165	0.122
	Most Popular	0.107	0.028	0.150	0.106	0.065
	User kNN	0.186	0.060	0.233	0.172	0.123
Unsupervised aggregation methods	CombMIN	0.120	0.031	0.129	0.125	0.083
	CombMED	0.149	0.042	0.192	0.144	0.098
	CombANZ	0.146	0.041	0.148	0.147	0.100
	LogISR	0.193 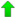	0.061	0.251	0.176	0.124
	Bordafuse	0.193 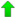	0.062	0.240	0.177	0.125
	Condorcet	0.178	0.057	0.246	0.161	0.114
	CombMAX	0.172	0.052	0.217	0.159	0.110
	CombSUM	0.184	0.058	0.241	0.166	0.116
	CombMNZ	0.192 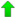	0.061	0.244	0.174	0.122
	ISR	0.187	0.059	0.247	0.169	0.119
Supervised aggregation methods	CombGMNZ	0.191	0.061	0.242	0.174	0.122
	RRF	0.193 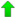	0.062	0.241	0.176	0.125
	Slidefuse	0.196 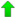	0.064 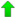	0.244	0.178 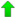	0.127
	Bayesfuse	0.195 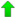	0.064 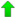	0.244	0.178 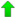	0.126
	WMNZ	0.184	0.060	0.238	0.164	0.121
	RBC	0.193 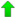	0.062	0.243	0.176	0.125
	LognISR	0.193 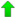	0.061	0.252	0.175	0.124
	Posfuse	0.196 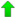	0.064 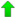	0.247	0.179 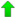	0.128 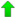
	Weighted Sum	0.183	0.058	0.227	0.169	0.121
	Weighted Borda	0.183	0.059	0.233	0.165	0.122

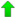
 Aggregation method is statistically better (*p* < 0.05), then all recommendation algorithms.

**Table 5:** The results of the experiments conducted on the MovieLens 1M dataset, representing the quality of recommendations generated by individual recommendation algorithms and aggregation methods.

Type	Algorithm Name	NDCG@10	MAP@10	P@1	P@10	Recall@10
Recommendation algorithms	BPR	0.113	0.027	0.146	0.116	0.065
	ImplicitMF	0.115	0.027	0.143	0.117	0.062
	Item kNN	0.125	0.030	0.168	0.127	0.063
	Most Popular	0.098	0.018	0.131	0.103	0.038
	User kNN	0.123	0.030	0.150	0.126	0.068
Unsupervised aggregation methods	CombMIN	0.105	0.022	0.117	0.114	0.058
	CombMED	0.116	0.025	0.134	0.122	0.063
	CombANZ	0.116	0.025	0.126	0.123	0.064
	LogISR	0.130 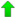	0.030	0.165	0.131 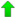	0.067
	Bordafuse	0.130 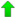	0.030	0.171	0.130 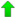	0.066
	Condorcet	0.116	0.026	0.159	0.117	0.058
	CombMAX	0.123	0.028	0.146	0.127	0.066
	CombSUM	0.129 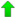	0.030	0.166	0.129	0.067
	CombMNZ	0.131 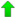	0.031	0.171	0.131 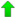	0.067
	ISR	0.129 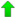	0.029	0.165	0.130	0.067
Supervised aggregation methods	CombGMNZ	0.131 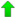	0.031	0.171	0.131 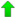	0.067
	RRF	0.130 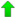	0.030	0.173	0.131 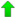	0.066
	Slidefuse	0.129 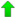	0.030	0.170	0.131 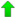	0.067
	Bayesfuse	0.129 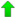	0.030	0.172	0.131 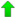	0.067
	WMNZ	0.129 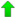	0.030	0.167	0.131 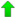	0.067
	RBC	0.130 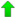	0.030	0.173	0.131 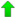	0.066
	LognISR	0.130 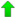	0.030	0.165	0.131 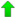	0.067
	Posfuse	0.130 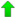	0.030	0.172	0.131 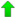	0.067
	Weighted Sum	0.129 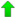	0.030	0.168	0.131 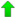	0.067
	Weighted Borda	0.125	0.030	0.167	0.127	0.063

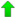
 Aggregation method is statistically better (*p* < 0.05), then all recommendation algorithms.

## Data Availability

The source code of RecRankAgg is publicly available via GitHub: https://github.com/mbalchanowski/RecRankAgg (accessed on 12 December 2022). Data used in experiments is available at: https://grouplens.org/datasets/movielens/100k/ (accessed on 12 December 2022) and https://grouplens.org/datasets/movielens/1m/ (accessed on 12 December 2022).

## Abbreviations

The following abbreviations are used in this manuscript:

*u*	Generic user
$u_{i}$	Specific user
$u_{A}$	Active user in system for which recommendations are generated
*U*	The set of all users
*i*	Generic item
$i_{j}$	Specific item
*I*	Set of all items
$a_{h}$	Specific recommendation algorithm
*A*	Set of *n* recommendation algorithms $A=\{a_{1},\;a_{2},\;\ldots ,\;a_{n}\}$
τ	Generic ranking
$\tau_{i}^{r}$	Ranking recommended to user $u_{i}$ by algorithm $a_{r}$ where r∈{1,2,…,n}
$\tau (i_{j})$	The position of item $i_{j}$ in ranking τ
*T*	Set of *n* rankings $T=\{\tau^{1},\tau^{2},\cdots ,\tau^{n}\}$

## References

[1] Bawden D., Robinson L. (2020). Information Overload: An Overview. Oxford Encyclopedia of Political Decision Making.

[2] Wani A., Joshi I., Khandve S., Wagh V., Joshi R. (2021). Evaluating Deep Learning Approaches for Covid19 Fake News Detection. Combating Online Hostile Posts in Regional Languages during Emergency Situation.

[3] Burke R., Felfernig A., Göker M.H. (2011). Recommender Systems: An Overview. AI Mag..

[4] Rafailidis D., Nanopoulos A. (2016). Modeling Users Preference Dynamics and Side Information in Recommender Systems. IEEE Trans. Syst. Man Cybern. Syst..

[5] Bennett J., Lanning S., Netflix N. The Netflix Prize. Proceedings of the KDD Cup and Workshop in Conjunction with KDD.

[6] Deshpande M., Karypis G. (2004). Item-Based Top-N Recommendation Algorithms. ACM Trans. Inf. Syst..

[7] Karatzoglou A., Baltrunas L., Shi Y. Learning to Rank for Recommender Systems. Proceedings of the 7th ACM Conference on Recommender Systems.

[8] Steck H. Evaluation of Recommendations: Rating-Prediction and Ranking. Proceedings of the 7th ACM Conference on Recommender Systems.

[9] Shani G., Gunawardana A. (2011). Evaluating recommendation systems. Recommender Systems Handbook.

[10] Anelli V.W., Bellogín A., Di Noia T., Jannach D., Pomo C. Top-N Recommendation Algorithms: A Quest for the State-of-the-Art. Proceedings of the 30th ACM Conference on User Modeling, Adaptation and Personalization.

[11] Aggarwal C.C. (2016). Advanced Topics in Recommender Systems. Recommender Systems: The Textbook.

[12] Oliveira S.E.L., Diniz V., Lacerda A., Merschmanm L., Pappa G.L. (2020). Is Rank Aggregation Effective in Recommender Systems? An Experimental Analysis. ACM Trans. Intell. Syst. Technol..

[13] Beel J., Breitinger C., Langer S., Lommatzsch A., Gipp B. (2016). Towards reproducibility in recommender-systems research. User Model. User-Adapt. Interact..

[14] Sun Z., Han L., Huang W., Wang X., Zeng X., Wang M., Yan H. (2015). Recommender systems based on social networks. J. Syst. Softw..

[15] Dacrema M.F., Boglio S., Cremonesi P., Jannach D. (2021). A Troubling Analysis of Reproducibility and Progress in Recommender Systems Research. ACM Trans. Inf. Syst..

[16] Cremonesi P., Jannach D. (2022). Progress in Recommender Systems Research: Crisis? What Crisis?. AI Mag..

[17] List C., Zalta E.N. (2022). Social Choice Theory. The Stanford Encyclopedia of Philosophy.

[18] Dwork C., Kumar R., Naor M., Sivakumar D. Rank Aggregation Methods for the Web. Proceedings of the 10th International Conference on World Wide Web.

[19] DeConde R.P., Hawley S., Falcon S., Clegg N., Knudsen B., Etzioni R. (2006). Combining Results of Microarray Experiments: A Rank Aggregation Approach. Stat. Appl. Genet. Mol. Biol..

[20] Fagin R., Kumar R., Sivakumar D. Efficient Similarity Search and Classification via Rank Aggregation. Proceedings of the 2003 ACM SIGMOD International Conference on Management of Data.

[21] Lin S. (2010). Rank aggregation methods. WIREs Comput. Stat..

[22] Smyth B., Cotter P. (2000). Personalized TV listings service for the digital TV age. Knowl.-Based Syst..

[23] Torres R., McNee S., Abel M., Konstan J., Riedl J. Enhancing digital libraries with TechLens. Proceedings of the 2004 Joint ACM/IEEE Conference on Digital Libraries.

[24] Boratto L., Carta S.A., Vargiu E., Armano G., Paddeu G., Soro A., Vargiu E., Armano G., Paddeu G. (2011). State-of-the-Art in Group Recommendation and New Approaches for Automatic Identification of Groups. Information Retrieval and Mining in Distributed Environments.

[25] Baltrunas L., Makcinskas T., Ricci F. (2010). Group Recommendations with Rank Aggregation and Collaborative Filtering. Proceedings of the Fourth ACM Conference on Recommender Systems (RecSys).

[26] Tang Y., Tong Q. BordaRank: A ranking aggregation based approach to collaborative filtering. Proceedings of the 2016 IEEE/ACIS 15th International Conference on Computer and Information Science (ICIS).

[27] Yalcin E., Ismailoglu F., Bilge A. (2021). An entropy empowered hybridized aggregation technique for group recommender systems. Expert Syst. Appl..

[28] Bartholdi J., Tovey C.A., Trick M.A. (1989). Voting Schemes for which It Can Be Difficult to Tell Who Won the Election. Soc. Choice Welf..

[29] Ribeiro M.T., Ziviani N., Moura E.S.D., Hata I., Lacerda A., Veloso A. (2015). Multiobjective Pareto-Efficient Approaches for Recommender Systems. ACM Trans. Intell. Syst. Technol..

[30] Oliveira S., Diniz V., Lacerda A., Pappa G.L. Evolutionary rank aggregation for recommender systems. Proceedings of the 2016 IEEE Congress on Evolutionary Computation (CEC).

[31] Oliveira S., Diniz V., Lacerda A., Pappa G.L. Multi-objective Evolutionary Rank Aggregation for Recommender Systems. Proceedings of the 2018 IEEE Congress on Evolutionary Computation (CEC).

[32] Bałchanowski M., Boryczka U. (2022). Aggregation of Rankings Using Metaheuristics in Recommendation Systems. Electronics.

[33] Ricci F., Rokach L., Shapira B.F., Rokach L., Shapira B., Ricci F., Rokach L., Shapira B. (2015). Recommender Systems: Introduction and Challenges. Recommender Systems Handbook.

[34] Bell R.M., Koren Y., Volinsky C. (2010). All Together Now: A Perspective on the Netflix Prize. Chance.

[35] Bell R.M., Koren Y., Volinsky C. (2007). The BellKor Solution to the Netflix Prize.

[36] Khatwani S., Chandak M. Building Personalized and Non Personalized recommendation systems. Proceedings of the 2016 International Conference on Automatic Control and Dynamic Optimization Techniques (ICACDOT).

[37] Pazzani M.J., Billsus D.P., Kobsa A., Nejdl W., Brusilovsky P., Kobsa A., Nejdl W. (2007). Content-Based Recommendation Systems. The Adaptive Web: Methods and Strategies of Web Personalization.

[38] Schafer J.B., Frankowski D., Herlocker J., Sen S. (2007). Collaborative Filtering Recommender Systems. The Adaptive Web.

[39] Aggarwal C.C. (2016). Knowledge-Based Recommender Systems. Recommender Systems: The Textbook.

[40] Çano E., Morisio M. (2017). Hybrid recommender systems: A systematic literature review. Intell. Data Anal..

[41] Koren Y., Bell R., Volinsky C. (2009). Matrix Factorization Techniques for Recommender Systems. Computer.

[42] Piatetsky-Shapiro G. (2007). Interview with Simon Funk. Sigkdd Explor..

[43] Ekstrand M.D. (2020). LensKit for Python: Next-Generation Software for Recommender Systems Experiments. Proceedings of the 29th ACM International Conference on Information & Knowledge Management (CIKM).

[44] Rendle S., Freudenthaler C., Gantner Z., Schmidt-Thieme L. (2009). BPR: Bayesian Personalized Ranking from Implicit Feedback. Proceedings of the Twenty-Fifth Conference on Uncertainty in Artificial Intelligence (UAI).

[45] Hu Y., Koren Y., Volinsky C. Collaborative Filtering for Implicit Feedback Datasets. Proceedings of the 2008 Eighth IEEE International Conference on Data Mining (ICDM).

[46] Klementiev A., Roth D., Small K. (2008). Unsupervised Rank Aggregation with Distance-Based Models. Proceedings of the 25th International Conference on Machine Learning (ICML).

[47] Liu Y.T., Liu T.Y., Qin T., Ma Z.M., Li H. (2007). Supervised Rank Aggregation. Proceedings of the 16th International Conference on World Wide Web (WWW).

[48] Liu T.Y. (2009). Learning to Rank for Information Retrieval. Found. Trends Inf. Retr..

[49] Li X., Wang X., Xiao G. (2017). A comparative study of rank aggregation methods for partial and top ranked lists in genomic applications. Briefings Bioinform..

[50] Fox E.A., Shaw J.A. (1993). Combination of Multiple Searches. In Proceedings of the TREC. https://trec.nist.gov/pubs/trec2/papers/txt/23.txt.

[51] Mourão A., Martins F., Magalhães J. (2015). Multimodal medical information retrieval with unsupervised rank fusion. Comput. Med. Imaging Graph..

[52] Aslam J.A., Montague M.H., Croft W.B., Harper D.J., Kraft D.H., Zobel J. (2001). Models for Metasearch. Proceedings of the 24th Annual International ACM SIGIR Conference on Research and Development in Information Retrieval.

[53] Montague M.H., Aslam J.A. (2002). Condorcet fusion for improved retrieval. Proceedings of the 2002 ACM CIKM International Conference on Information and Knowledge Management.

[54] Lee J.H. (1997). Analyses of Multiple Evidence Combination. Proceedings of the 20th Annual International ACM SIGIR Conference on Research and Development in Information Retrieval.

[55] Cormack G.V., Clarke C.L.A., Buettcher S. (2009). Reciprocal Rank Fusion Outperforms Condorcet and Individual Rank Learning Methods. Proceedings of the 32nd International ACM SIGIR Conference on Research and Development in Information Retrieval.

[56] Lillis D., Toolan F., Collier R.W., Dunnion J., Macdonald C., Ounis I., Plachouras V., Ruthven I., White R.W. (2008). Extending Probabilistic Data Fusion Using Sliding Windows. Proceedings of the Advances in Information Retrieval, 30th European Conference on IR Research.

[57] Wu S., Crestani F. (2002). Data fusion with estimated weights. Proceedings of the 2002 ACM CIKM International Conference on Information and Knowledge Management.

[58] Bailey P., Moffat A., Scholer F., Thomas P., Kando N., Sakai T., Joho H., Li H., de Vries A.P., White R.W. (2017). Retrieval Consistency in the Presence of Query Variations. Proceedings of the 40th International ACM SIGIR Conference on Research and Development in Information Retrieval.

[59] Lillis D., Zhang L., Toolan F., Collier R.W., Leonard D., Dunnion J., Crestani F., Marchand-Maillet S., Chen H., Efthimiadis E.N., Savoy J. (2010). Estimating probabilities for effective data fusion. Proceedings of the Proceeding of the 33rd International ACM SIGIR Conference on Research and Development in Information Retrieval.

[60] Bassani E. (2022). ranx: A Blazing-Fast Python Library for Ranking Evaluation and Comparison. Proceedings of the European Conference on Information Retrieval (ECIR).

[61] Bassani E., Romelli L. (2022). ranx.fuse: A Python Library for Metasearch. Proceedings of the 31st ACM International Conference on Information and Knowledge Management (CIKM).

[62] Harper F.M., Konstan J.A. (2015). The MovieLens Datasets: History and Context. ACM Trans. Interact. Intell. Syst..

[63] Akiba T., Sano S., Yanase T., Ohta T., Koyama M. Optuna: A Next-generation Hyperparameter Optimization Framework. Proceedings of the 25rd ACM SIGKDD International Conference on Knowledge Discovery and Data Mining.

[64] Bergstra J., Bardenet R., Bengio Y., Kégl B., Shawe-Taylor J., Zemel R., Bartlett P., Pereira F., Weinberger K. (2011). Algorithms for Hyper-Parameter Optimization. Proceedings of the Advances in Neural Information Processing Systems (NIPS).

[65] Smucker M.D., Allan J., Carterette B. (2007). A Comparison of Statistical Significance Tests for Information Retrieval Evaluation. Proceedings of the Sixteenth ACM Conference on Conference on Information and Knowledge Management (CIKM).

[66] Lin Z., Li Y., Guo X. (2017). Consensus measure of rankings. arXiv.

[67] Asudeh A., Jagadish H.V., Stoyanovich J., Das G. Designing Fair Ranking Schemes. Proceedings of the 2019 International Conference on Management of Data.

[68] Kuhlman C., Rundensteiner E. (2020). Rank Aggregation Algorithms for Fair Consensus. Proc. VLDB Endow..

